# Supplementary material for: Multidimensional cognitive deficits in the typical and atypical variants of Alzheimer’s disease
Source: Alzheimers Res Ther. 2025 Oct 16;17:226. doi: 10.1186/s13195-025-01873-w (PMC12532852; doi:10.1186/s13195-025-01873-w)
Supplement: Supplementary file 1 — Supplementary Material 1. [file 13195_2025_1873_MOESM1_ESM.docx]

**Supplementary material**

**Supplementary Table 1 Loadings for neuropsychological battery principal component analysis**

| **Measure** | **PC 1 (“Multi-domain cognition”)** | **PC 2 (“Non-verbal memory”)** |
| --- | --- | --- |
| CSB Naming | **0.70** | -0.10 |
| CAT Comprehension of Spoken Sentences | **0.89** | 0.00 |
| Trail Making Test B | **0.84** | 0.32 |
| Brixton | **0.52** | 0.20 |
| Raven’s Coloured Progressive Matrices | **0.61** | 0.26 |
| Digit Span Backward | 0.40 | 0.22 |
| RAVLT Immediate Recall | **0.74** | 0.17 |
| RAVLT Delayed Recall | **0.69** | 0.14 |
| ROCF Immediate Recall | 0.13 | **0.98** |
| ROCF Delayed Recall | 0.17 | **0.96** |

Rotation: Orthogonal varimax. Loadings above a threshold of 0.5 are bolded. PC, principal component; TTR, type-to-token ratio. CAT, Comprehensive Aphasia Test; CSB, Cambridge Semantic Battery; RAVLT, Rey Auditory Verbal Learning Test; ROCF, Rey-Osterrieth Figure Copy.

**Supplementary Table 2 Verbal and non-verbal episodic memory tests *post hoc* group comparisons**

| **Task** | **Phase or condition** | **Post hoc *t*-tests** |
| --- | --- | --- |
| Rey Auditory Verbal Learning Test (RAVLT) | Early learning | Controls > lvPPA+ (*t* = 6.06, *P* < 0.001, BF > 100)  Controls > lvPPA (*t* = 4.40, *P* < 0.001, BF = 86.34)  Controls > tAD (*t* = 4.03, *P* < 0.001, BF = 36.18)  tAD > lvPPA+ (*t* = 3.01, *P* = 0.01, BF = 4.61)  Either no or anecdotal evidence between other patient group comparisons (0.33 < BF < 3) |
|  | Late learning | Controls > lvPPA+ (*t* = 8.63, *P* < 0.001, BF > 100)  Controls > tAD (*t* = 5.43, *P* < 0.001, BF > 100)  Controls > lvPPA (*t* = 4.66, *P* < 0.001, BF > 100)  Either no or anecdotal evidence between all patient group comparisons (0.33 < BF < 3) |
|  | Immediate recall | Controls > lvPPA+ (*t* = 6.08, *P* < 0.001, BF > 100)  Controls > tAD (*t* = 3.62, *P* = 0.002, BF = 17.62)  Controls > lvPPA (*t* = 3.06, *P* = 0.006, BF = 7.37)  Either no or anecdotal evidence between all patient group comparisons (0.33 < BF < 3) |
|  | Delayed recall | Controls > lvPPA+ (*t* = 4.63, *P* < 0.001, BF = 67.71)  Controls > tAD (*t* = 3.67, *P* = 0.002, BF = 19.00))  Controls > lvPPA (*t* = 2.59, *P* = 0.02, BF = 3.36)  Either no or anecdotal evidence between all patient group comparisons (0.33 < BF < 3) |
|  | Recognition true positive | Either no or anecdotal evidence between all group comparisons (0.33 < BF < 3) |
|  | Recognition false positive | Controls > lvPPA+ (*t* = -4.81, *P* < 0.001, BF = 90.40)  Controls > tAD (*t* = -3.54, *P* = 0.002, BF = 15.24)  lvPPA > lvPPA+ (*t* = -3.12, *P* = 0.009, BF = 5.57)  Either no or anecdotal evidence between all other group comparisons (0.33 < BF < 3) |
| Rey Osterrieth complex figure (ROCF) | Copy | Controls > lvPPA+ (*t* = 3.49, *P* = 0.003, BF = 14.09)  Controls > lvPPA (*t* = 3.29, *P* = 0.004, BF = 10.51)  Controls > tAD (*t* = 2.62, *P* = 0.02, BF = 3.56)  Either no or anecdotal evidence between all patient group comparisons (0.33 < BF < 3) |
|  | Immediate recall | Controls > lvPPA+ (*t* = 6.87, *P* < 0.001, BF > 100)  Controls > tAD (*t* = 4.81, *P* < 0.001, BF > 100)  lvPPA > lvPPA+ (*t* = 4.08, *P* < 0.001, BF = 29.61)  Either no or anecdotal evidence between all other group comparisons (0.33 < BF < 3) |
|  | Delayed recall | Controls > lvPPA+ (*t* = 7.31, *P* < 0.001, BF > 100)  Controls > tAD (*t* = 4.91, *P* < 0.001, BF > 100)  Controls > lvPPA (*t* = 2.66, *P* = 0.02, BF = 3.79)  lvPPA > lvPPA+ (*t* = 3.84, *P* = 0.002, BF = 20.49)  Either no or anecdotal evidence between all other group comparisons (0.33 < BF < 3) |
|  | Recognition true positive | Either no or anecdotal evidence between all group comparisons (0.33 < BF < 3) |
|  | Recognition false positive | Controls > lvPPA+ (*t* = -5.27, *P* < 0.001, BF > 100)  Controls > tAD (*t* = -4.36, *P* < 0.001, BF = 64.40)  Controls > lvPPA (*t* = -4.16, *P* < 0.001, BF = 49.82)  No evidence between all patient group comparisons (0.33 < BF < 1) |

Note: The direction of the “greater than” symbol or chevron (>) indicates the group with better performance. For example, for the “late learning” phase of the Rey Auditory Verbal Learning Test, evidence for controls performing better (i.e., higher scores) was extreme relative to all patient groups. For the “false positive” condition of recognition trials, lower scores (i.e., less errors) indicate better performance.

**Supplementary Table 3 Bayesian point and interval estimates of effect sizes for each longitudinal case to group sample**

| Participant | Measure (maximum score, if applicable) | Group mean* (SD) | Group N | Initial assessment score/ value | Bayesian two-tailed probability that a member of the group sample would obtain a lower score than the case | Bayesian point estimate of percentage of group sample falling below case’s score (95% credible interval) | Bayesian effect size of percentage of group sample falling below the case’s score (95% credible interval) | Follow-up assessment score or value | Bayesian two-tailed probability | Bayesian point estimate (95% credible interval) | Bayesian effect size (95% credible interval) |
| --- | --- | --- | --- | --- | --- | --- | --- | --- | --- | --- | --- |
| **Principal Component (PC) Scores** | | | | | | | | | | | |
| tAD 1 | PC 1 | 0.72 (0.69) | 9 | 1.84 | 0.16 | 91.89 (72.01 to 99.56) | 1.62 (0.58 to 2.62) | 2.25 | 0.07 | 96.57 (82.82 to 99.97) | 2.22 (0.95 to 3.45) |
| tAD 2 |  |  |  | 0.17 | 0.47 | 23.57 (6.24 to 49.14) | -0.80 (-1.54 to -0.02) | -0.23 | 0.23 | 11.40 (1.12 to 33.49) | -1.38 (-2.29 to -0.43) |
| tAD 3 |  |  |  | 0.96 | 0.75 | 62.51 (36.81 to 84.43) | 0.35 (-0.34 to 1.01) | -0.69 | 0.09 | 4.43 (0.07 to 19.87) | -2.04 (-3.20 to -0.85) |
| lvPPA 1 |  | 0.24 (0.68) | 10 | 0.42 | 0.81 | 59.69 (35.45 to 81.31) | 0.27 (-0.37 to 0.89) | -0.50 | 0.33 | 16.34 (3.13 to 39.10) | -1.09 (-1.86 to -0.28) |
| lvPPA 2 |  |  |  | 0.52 | 0.70 | 64.82 (40.28 to 85.28) | 0.41 (-0.25 to 1.05) | -1.38 | **0.05** | 2.46 (0.02 to 13.10) | -2.38 (-3.61 to -1.12) |
| lvPPA+ 1 |  | -1.11 (0.65) | 8 | -1.30 | 0.79 | 39.56 (16.07 to 66.53) | -0.29 (-0.99 to 0.43) | -2.02 | 0.23 | 11.43 (0.88 to 35.21) | -1.40 (-2.37 to -0.38) |
| lvPPA+ 2 |  |  |  | -0.69 | 0.56 | 71.93 (44.38 to 91.86) | 0.65 (-0.14 to 1.40) | -1.48 | 0.61 | 30.42 (9.63 to 57.94) | -0.57 (-1.30 to 0.20) |
| tAD 1 | PC 2 | -0.30 (0.93) | 9 | 0.86 | 0.27 | 86.46 (63.23 to 98.27) | 1.25 (0.34 to 2.11) | 1.76 | 0.07 | 96.55 (82.78 to 99.997) | 2.22 (0.95 to 3.45) |
| tAD 2 |  |  |  | 0.38 | 0.51 | 75.62 (48.84 to 92.71) | 0.73 (-0.03 to 1.46) | -0.14 | 0.87 | 56.29 (31.16 to 79.56) | 0.17 (-0.49 to 0.83) |
| tAD 3 |  |  |  | -0.70 | 0.69 | 34.71 (13.52 to 60.56) | -0.43 (-1.10 to 0.27) | -0.22 | 0.94 | 53.16 (28.41 to 77.00) | 0.09 (-0.57 to 0.74) |
| lvPPA 1 |  | 0.66 (1.08) | 10 | 1.52 | 0.47 | 76.65 (52.46 to 93.28) | 0.80 (0.06 to 1.50) | 0.99 | 0.78 | 61.14 (36.80 to 82.45) | 0.31 (-0.34 to 0.93) |
| lvPPA 2 |  |  |  | 1.12 | 0.69 | 65.30 (40.74 to 85.64) | 0.43 (-0.23 to 1.06) | -0.43 | 0.36 | 18.06 (3.91 to 41.29) | -1.01 (-1.76 to -0.22) |
| lvPPA+ 1 |  | -0.49 (0.47) | 8 | 0.40 | 0.12 | 94.13 (75.12 to 99.89) | 1.89 (0.68 to 3.07) | -0.77 | 0.59 | 29.61 (9.10 to 57.12) | -0.60 (-1.33 to 0.18) |
| lvPPA+ 2 |  |  |  | -0.05 | 0.41 | 79.67 (52.80 to 96.05) | 0.94 (0.07 to 1.76) | -0.53 | 0.94 | 46.93 (21.89 to 73.02) | -0.09 (-0.78 to 0.61) |
| **ACE-R Sub-scores** | | | | | | | | | | | |
| tAD 1 | ACE-R language (26) | 25.00 (1.12) | 9 | 26 | 0.42 | 78.92 (53.62 to 95.10) | 0.89 (0.09 to 1.66) | 26 | 0.42 | 78.92 (53.62 to 95.10) | 0.89 (0.09 to 1.66) |
| tAD 2 |  |  |  | 25 | 1.00 | 50.01 (25.69 to 74.34) | 0.00 (-0.65 to 0.65) | 25 | 1.00 | 50.01 (25.69 to 74.34) | 0.00 (-0.65 to 0.65) |
| tAD 3 |  |  |  | 26 | 0.42 | 78.92 (53.62 to 95.10) | 0.89 (0.09 to 1.66) | 12 | **<0.001** | 0.0002 (0.00 to 0.00) | -11.61 (-17.22 to -6.02) |
| lvPPA 1 |  | 16.60 (3.98) | 10 | 14 | 0.55 | 27.45 (9.26 to 51.97) | -0.65 (-1.33 to 0.05) | 2 | **0.007** | 0.34 (0.00 to 2.98) | -3.78 (-5.43 to -1.88) |
| lvPPA 2 |  |  |  | 22 | 0.23 | 88.60 (67.84 to 98.65) | 1.36 (0.46 to 2.21) | 7 | **0.05** | 2.35 (0.01 to 12.72) | -2.41 (-3.65 to -1.14) |
| lvPPA+ 1 |  | 12.70 (5.85) | 7 | 6 | 0.33 | 16.26 (1.92 to 44.18) | -1.15 (-2.09 to -0.15) | 2 | 0.14 | 6.90 (0.11 to 28.91) | -1.83 (-3.06 to -0.56) |
| tAD 1 | ACE-R memory (26) | 15.60 (4.42) | 9 | 25 | 0.08 | 96.08 (81.40 to 99.96) | 2.13 (0.89 to 3.32) | 26 | 0.06 | 97.19 (84.78 to 99.99) | 2.35 (1.03 to 3.64) |
| tAD 2 |  |  |  | 15 | 0.90 | 45.04 (21.56 to 70.04) | -0.14 (-0.79 to 0.53) | 9 | 0.19 | 9.72 (0.73 to 30.77) | -1.49 (-2.44 to -0.50) |
| tAD 3 |  |  |  | 12 | 0.46 | 23.11 (5.98 to 48.63) | -0.81 (-1.56 to -0.03) | 10 | 0.26 | 13.20 (1.63 to 36.19) | -1.27 (-2.14 to -0.35) |
| lvPPA 1 |  | 12.90 (4.51) | 10 | 11 | 0.70 | 34.88 (14.50 to 59.44) | -0.42 (-1.06 to 0.24) | 2 | **0.05** | 2.33 (0.01 to 12.66) | -2.42 (-3.66 to -1.14) |
| lvPPA 2 |  |  |  | 17 | 0.41 | 79.57 (55.81 to 94.90) | 0.91 (0.15 to 1.64) | 8 | 0.33 | 16.37 (3.15 to 39.15) | -1.09 (-1.86 to -0.28) |
| lvPPA+ 1 |  | 5.55 (4.10) | 7 | 2 | 0.45 | 22.45 (2.26 to 51.57) | -0.87 (-1.72 to 0.04) | 0 | 0.25 | 12.62 (0.87 to 39.08) | -1.35 (-2.38 to -0.28) |
| tAD 1 | ACE-R visuospatial (16) | 13.40 (2.55) | 9 | 15 | 0.57 | 71.59 (45.65 to 90.83) | 0.63 (-0.11 to 1.33) | 16 | 0.36 | 81.91 (57.17 to 96.53) | 1.02 (0.18 to 1.82) |
| tAD 2 |  |  |  | 16 | 0.36 | 81.91 (57.17 to 96.53) | 1.02 (0.18 to 1.82) | 11 | 0.40 | 19.91 (4.32 to 44.94) | -0.94 (-1.72 to -0.13) |
| tAD 3 |  |  |  | 15 | 0.57 | 71.59 (45.65 to 90.83) | 0.63 (-0.11 to 1.33) | 12 | 0.62 | 30.84 (10.79 to 56.79) | -0.55 (-1.24 to 0.17) |
| lvPPA 1 |  | 12.70 (2.00) | 10 | 15 | 0.30 | 84.93 (62.53 to 97.39) | 1.15 (0.32 to 1.94) | 14 | 0.55 | 72.46 (47.93 to 90.67) | 0.65 (-0.05 to 1.32) |
| lvPPA 2 |  |  |  | 14 | 0.55 | 72.46 (47.93 to 90.67) | 0.65 (-0.05 to 1.32) | 9 | 0.11 | 5.58 (0.20 to 21.51) | -1.85 (-2.88 to -0.79) |
| lvPPA+ 1 |  | 9.28 (4.31) | 7 | 9 | 0.95 | 47.69 (21.08 to 75.17) | -0.07 (-0.80 to 0.68) | 6 | 0.50 | 25.17 (5.61 to 54.52) | -0.76 (-1.59 to 0.11) |
| **Language Scores** | | | | | | | | | | | |
| tAD 1 | CSB naming (64) | 61.90 (2.32) | 9 | 64 | 0.42 | 79.22 (53.97 to 95.26) | 0.91 (0.10 to 1.67) | 64 | 0.42 | 79.22 (53.97 to 95.26) | 0.91 (0.10 to 1.67) |
| tAD 2 |  |  |  | 64 | 0.42 | 79.22 (53.97 to 95.26) | 0.91 (0.10 to 1.67) | 64 | 0.42 | 79.22 (53.97 to 95.26) | 0.91 (0.10 to 1.67) |
| tAD 3 |  |  |  | 57 | 0.08 | 4.01 (0.05 to 18.72) | -2.11 (-3.30 to -0.89) | 26 | **<0.001** | 0.00 (0.00 to 0.00) | -15.47 (-22.93 to -8.05) |
| lvPPA 1 |  | 49.00 (11.70) | 10 | 48 | 0.94 | 46.85 (24.09 to 70.49) | -0.09 (-0.70 to 0.54) | 22 | 0.06 | 2.77 (0.02 to 14.10) | -2.31 (-3.51 to -1.08) |
| lvPPA 2 |  |  |  | 54 | 0.69 | 65.35 (40.79 to 85.67) | 0.43 (-0.23 to 1.07) | 34 | 0.25 | 12.64 (1.73 to 33.98) | -1.28 (-2.11 to -0.41) |
| lvPPA+ 1 |  | 23.90 (17.10) | 8 | 1 | 0.25 | 12.37 (1.10 to 36.66) | -1.34 (-2.29 to -0.34) | 0 | 0.23 | 11.46 (0.89 to 35.27) | -1.40 (-2.37 to -0.38) |
| lvPPA+ 2 |  |  |  | 10 | 0.47 | 23.44 (5.48 to 50.67) | -0.81 (-1.60 to 0.02) | 0 | 0.23 | 11.46 (0.89 to 35.27) | -1.40 (-2.37 to -0.38) |
| tAD 1 | ACE-R repetition (6) | 5.56 (0.72) | 9 | 6 | 0.58 | 71.10 (45.15 to 90.51) | 0.61 (-0.12 to 1.31) | 6 | 0.58 | 71.10 (45.15 to 90.51) | 0.61 (-0.12 to 1.31) |
| tAD 2 |  |  |  | 5 | 0.48 | 24.09 (6.53 to 49.72) | -0.78 (-1.51 to -0.01) | 5 | 0.48 | 24.09 (6.53 to 49.72) | -0.78 (-1.51 to -0.01) |
| tAD 3 |  |  |  | 6 | 0.58 | 71.10 (45.15 to 90.51) | 0.61 (-0.12 to 1.31) | 5 | 0.48 | 24.09 (6.53 to 49.72) | -0.78 (-1.51 to -0.01) |
| lvPPA 1 |  | 1.90 (2.02) | 10 | 2 | 0.96 | 51.84 (28.39 to 74.83) | 0.05 (-0.57 to 0.67) | 1 | 0.68 | 34.06 (13.89 to 58.65) | -0.45 (-1.09 to 0.22) |
| lvPPA 2 |  |  |  | 3 | 0.62 | 69.20 (44.57 to 88.46) | 0.55 (-0.14 to 1.20) | 0 | 0.39 | 19.67 (4.70 to 43.25) | -0.94 (-1.68 to -0.17) |
| lvPPA+ 1 |  | 2.83 (1.76) | 7 | 2 | 0.67 | 33.74 (10.77 to 62.95) | -0.47 (-1.24 to 0.33) | 2 | 0.67 | 33.74 (10.77 to 62.95) | -0.47 (-1.24 to 0.33) |
| tAD 1 | CAT comprehension of spoken sentences (32) | 27.80 (2.73) | 9 | 30 | 0.47 | 76.67 (51.09 to 93.90) | 0.81 (0.03 to 1.55) | 28 | 0.95 | 52.69 (28.01 to 76.62) | 0.07 (-0.58 to 0.73) |
| tAD 2 |  |  |  | 28 | 0.95 | 52.69 (28.01 to 76.62) | 0.07 (-0.58 to 0.73) | 26 | 0.55 | 27.46 (8.57 to 53.35) | -0.66 (-1.37 to 0.08) |
| tAD 3 |  |  |  | 30 | 0.47 | 76.67 (51.09 to 93.90) | 0.81 (0.03 to 1.55) | 14 | **0.001** | 0.07 (0.00 to 0.55) | -5.06 (-7.56 to -2.54) |
| lvPPA 1 |  | 24.80 (4.13) | 10 | 28 | 0.48 | 76.06 (51.81 to 92.93) | 0.78 (0.05 to 1.47) | 26 | 0.79 | 60.61 (36.31 to 82.04) | 0.29 (-0.35 to 0.92) |
| lvPPA 2 |  |  |  | 24 | 0.86 | 42.89 (20.79 to 66.94) | -0.19 (-0.81 to 0.44) | 22 | 0.53 | 26.72 (8.79 to 51.19) | -0.68 (-1.35 to 0.03) |
| lvPPA+ 1 |  | 18.20 (4.71) | 8 | 18 | 0.97 | 48.47 (23.16 to 74.33) | -0.04 (-0.73 to 0.65) | 14 | 0.43 | 21.43 (4.47 to 48.40) | -0.89 (-1.70 to -0.04) |
| lvPPA+ 2 |  |  |  | 24 | 0.28 | 85.82 (60.63 to 98.40) | 1.23 (0.27 to 2.15) | 16 | 0.67 | 33.66 (11.81 to 61.09) | -0.47 (-1.19 to 0.28) |
| **Executive/visuospatial Scores** | | | | | | | | | | | |
| tAD 1 | Trail Making Test A | 0.34 (0.17) | 9 | 0.48 | 0.46 | 77.14 (51.60 to 94.16) | 0.82 (0.04 to 1.57) | 0.98 | **0.007** | 99.63 (96.64 to 100.00) | 3.77 (1.83 5.67) |
| tAD 2 |  |  |  | 0.48 | 0.46 | 77.14 (51.60 to 94.16) | 0.82 (0.04 to 1.57) | 0.22 | 0.52 | 26.11 (7.72 to 51.92) | -0.71 (-1.42 to 0.05) |
| tAD 3 |  |  |  | 0.48 | 0.46 | 77.14 (51.60 to 94.16) | 0.82 (0.04 to 1.57) | 0.30 | 0.83 | 41.46 (18.66 to 66.84) | -0.24 (-0.89 to 0.44) |
| lvPPA 1 |  | 0.41 (0.22) | 10 | 0.55 | 0.56 | 72.05 (47.50 to 90.40) | 0.64 (-0.06 to 1.31) | 0.39 | 0.93 | 46.65 (23.92 to 70.31) | -0.09 (-0.71 to 0.53) |
| lvPPA 2 |  |  |  | 0.19 | 0.37 | 18.27 (4.04 to 41.55) | -1.00 (-1.75 to -0.21) | 0.02 | 0.13 | 6.27 (0.28 to 22.99) | -1.77 (-2.77 to -0.74) |
| lvPPA+ 1 |  | 0.17 (0.19) | 7 | 0.10 | 0.74 | 37.12 (13.08 to 66.03) | -0.37 (-1.12 to 0.41) | 0.01 | 0.46 | 23.05 (4.54 to 52.22) | -0.84 (-1.69 to 0.06) |
| lvPPA+ 2 |  |  |  | 0.20 | 0.89 | 55.64 (27.62 to 81.55) | 0.16 (-0.59 to 0.90) | 0.12 | 0.81 | 40.70 (15.67 to 69.23) | -0.26 (-1.01 to 0.50) |
| tAD 1 | Digit span forward (12) | 8.67 (2.00) | 9 | 12 | 0.15 | 92.35 (72.89 to 99.63) | 1.67 (0.61 to 2.68) | 11 | 0.30 | 84.94 (61.09 to 97.75) | 1.17 (0.28 to 2.00) |
| tAD 2 |  |  |  | 7 | 0.45 | 22.57 (5.68 to 48.03) | -0.84 (-1.58 to -0.05) | 5 | 0.12 | 6.00 (0.18 to 23.61) | -1.84 (-2.91 to -0.72) |
| tAD 3 |  |  |  | 10 | 0.55 | 72.71 (46.81 to 91.55) | 0.67 (-0.08 to 1.38) | 7 | 0.45 | 22.57 (5.68 to 48.03) | -0.84 (-1.58 to -0.05) |
| lvPPA 1 |  | 4.90 (2.42) | 10 | 5 | 0.97 | 51.54 (28.12 to 64.57) | 0.04 (-0.58 to 0.66) | 1 | 0.16 | 7.95 (0.54 to 26.29) | -1.61 (-2.55 to -0.63) |
| lvPPA 2 |  |  |  | 5 | 0.97 | 51.54 (28.12 to 64.57) | 0.04 (-0.58 to 0.66) | 4 | 0.73 | 36.57 (15.77 to 61.05) | -0.37 (-1.00 to 0.28) |
| lvPPA+ 1 |  | 4.88 (3.09) | 8 | 6 | 0.74 | 62.88 (35.69 to 85.75) | 0.36 (-0.37 to 1.07) | 4 | 0.80 | 39.82 (16.27 to 66.77) | -0.29 (-0.98 to 0.43) |
| lvPPA+ 2 |  |  |  | 3 | 0.58 | 29.22 (8.86 to 56.74) | -0.61 (-1.35 to 0.17) | 0 | 0.18 | 9.01 (0.44 to 31.16) | -1.58 (-2.62 to -0.49) |
| tAD 1 | Digit span backward (12) | 4.44 (1.81) | 9 | 7 | 0.22 | 89.17 (67.33 to 99.02) | 1.41 (0.45 to 2.34) | 11 | **0.009** | 99.56 (96.02 to 100.00) | 3.62 (1.75 to 5.47) |
| tAD 2 |  |  |  | 5 | 0.78 | 61.17 (35.58 to 83.41) | 0.31 (-0.37 to 0.97) | 4 | 0.82 | 41.18 (18.44 to 66.59) | -0.24 (-0.90 to 0.43) |
| tAD 3 |  |  |  | 4 | 0.82 | 41.18 (18.44 to 66.59) | -0.24 (-0.90 to 0.43) | 2 | 0.24 | 11.85 (1.23 to 34.18) | -1.35 (-2.25 to -0.41) |
| lvPPA 1 |  | 3.30 (1.64) | 10 | 5 | 0.35 | 82.56 (59.44 to 96.37) | 1.04 (0.24 to 1.80) | 2 | 0.47 | 23.47 (6.79 to 47.64) | -0.79 (-1.49 to -0.06) |
| lvPPA 2 |  |  |  | 3 | 0.87 | 43.28 (21.12 to 67.30) | -0.18 (-0.80 to 0.45) | 2 | 0.47 | 23.47 (6.79 to 47.64) | -0.79 (-1.49 to -0.06) |
| lvPPA+ 1 |  | 2.75 (1.91) | 8 | 3 | 0.90 | 54.75 (28.46 to 79.50) | 0.13 (-0.57 to 0.82) | 2 | 0.72 | 36.12 (13.54 to 63.39) | -0.39 (-1.10 to 0.34) |
| lvPPA+ 2 |  |  |  | 1 | 0.42 | 20.83 (4.18 to 47.71) | -0.92 (-1.73 to -0.06) | 0 | 0.22 | 10.85 (0.76 to 34.28) | -1.44 (-2.43 to -0.41) |
| tAD 1 | Raven’s Coloured Progressive Matrices (12) | 9.56 (1.67) | 9 | 12 | 0.20 | 89.84 (68.41 to 99.18) | 1.46 (0.48 to 2.40) | 11 | 0.44 | 78.15 (52.74 to 94.70) | 0.86 (0.07 to 1.62) |
| tAD 2 |  |  |  | 9 | 0.76 | 37.94 (15.92 to 63.59) | -0.33 (-1.00 to 0.35) | 7 | 0.18 | 9.20 (0.63 to 29.88) | -1.53 (-2.50 -0.53) |
| tAD 3 |  |  |  | 11 | 0.44 | 78.15 (52.74 to 94.70) | 0.86 (0.07 to 1.62) | 10 | 0.81 | 59.56 (34.11 to 82.16) | 0.26 (-0.41 to 0.92) |
| lvPPA 1 |  | 8.85 (2.43) | 9 | 9 | 0.95 | 52.27 (27.63 to 76.26) | 0.06 (-0.59 to 0.72) | 11 | 0.43 | 78.72 (53.38 to 95.00) | 0.89 (0.09 to 1.64) |
| lvPPA 2 |  |  |  | 8 | 0.75 | 37.44 (15.54 to 63.13) | -0.35 (-1.01 to 0.34) | 2 | **0.03** | 1.41 (0.00 to 9.63) | -2.82 (-4.31 to -1.30) |
| lvPPA+ 1 |  | 6.43 (1.99) | 7 | 9 | 0.27 | 86.38 (59.34 to 98.91) | 1.29 (0.24 to 2.29) | 10 | 0.14 | 92.78 (70.32 to 99.87) | 1.79 (0.53 to 3.01) |
| lvPPA+ 2 |  |  |  | 8 | 0.49 | 75.59 (46.19 to 94.78) | 0.79 (-0.10 to 1.62) | 9 | 0.27 | 86.38 (59.34 to 98.91) | 1.29 (0.24 to 2.29) |
| **Memory Scores** | | | | | | | | | | | |
| tAD 1 | RAVLT “Early Learning” (15) | 3.64 (1.31) | 8 | 5.5 | 0.22 | 88.87 (65.20 to 99.18) | 1.42 (0.39 to 2.40) | 6.5 | 0.08 | 96.07 (80.12 to 99.97) | 2.18 (0.85 to 3.48) |
| tAD 2 |  |  |  | 3.5 | 0.92 | 46.14 (21.25 to 72.35) | -0.11 (-0.80 to 0.59) | 3 | 0.66 | 32.97 (11.33 to 60.43) | -0.49 (-1.21 to 0.27) |
| tAD 3 |  |  |  | 4.5 | 0.56 | 72.23 (44.70 to 92.05) | 0.66 (-0.13 to 1.41) | 3 | 0.66 | 32.97 (11.33 to 60.43) | -0.49 (-1.21 to 0.27) |
| lvPPA 1 |  | 3.30 (1.83) | 9 | 2 | 0.52 | 25.98 (7.65 to 51.78) | -0.71 (-1.43 to 0.05) | 1 | 0.27 | 13.37 (1.68 to 36.44) | -1.26 (-2.13 to -0.35) |
| tAD 1 | RAVLT “Late Learning” (15) | 4.56 (3.13) | 8 | 7.5 | 0.41 | 79.74 (52.89 to 96.08) | 0.94 (0.07 to 1.76) | 11 | 0.09 | 95.32 (78.04 to 99.95) | 2.06 (0.77 to 3.30) |
| tAD 2 |  |  |  | 3.5 | 0.76 | 37.96 (14.89 to 65.07) | -0.34 (-1.04 to 0.39) | 4 | 0.87 | 43.56 (19.19 to 70.10) | -0.18 (-0.87 to 0.53) |
| tAD 3 |  |  |  | 4 | 0.87 | 43.56 (19.19 to 70.10) | -0.18 (-0.87 to 0.53) | 3.5 | 0.76 | 37.96 (14.89 to 65.07) | -0.34 (-1.04 to 0.39) |
| lvPPA 1 |  | 5.85 (3.33) | 9 | 3.5 | 0.52 | 26.11 (7.73 to 51.92) | -0.71 (-1.42 to 0.05) | 1.5 | 0.25 | 12.53 (1.43 to 35.21) | -1.31 (-2.19 to -0.38) |
| tAD 1 | RAVLT Immediate Recall (15) | 5.46 (3.53) | 8 | 8 | 0.52 | 75.04 (46.57 to 93.12) | 0.72 (-0.09 to 1.49) | 10 | 0.26 | 86.77 (62.00 to 98.68) | 1.29 (0.31 to 2.22) |
| tAD 2 |  |  |  | 4 | 0.71 | 35.42 (13.04 to 62.74) | -0.41 (-1.12 to 0.33) | 3 | 0.53 | 26.62 (7.27 to 54.09) | -0.70 (-1.46 to 0.10) |
| tAD 3 |  |  |  | 5 | 0.91 | 45.30 (20.58 to 71.61) | -0.13 (-0.82 to 0.57) | 3 | 0.53 | 26.62 (7.27 to 54.09) | -0.70 (-1.46 to 0.10) |
| lvPPA 1 |  | 6.60 (4.01) | 9 | 6 | 0.89 | 44.54 (21.14 to 69.59) | -0.15 (-0.80 to 0.51) | 3 | 0.42 | 20.97 (4.84 to 46.19) | -0.90 (-1.66 to -0.10) |
| tAD 1 | RAVLT Delayed Recall (15) | 3.10 (3.37) | 8 | 4 | 0.81 | 59.59 (32.72 to 83.28) | 0.27 (-0.45 to 0.97) | 10 | 0.09 | 95.25 (77.86 to 99.95) | 2.05 (0.77 to 3.28) |
| tAD 2 |  |  |  | 0 | 0.41 | 20.74 (4.14 to 47.61) | -0.92 (-1.73 to -0.06) | 0 | 0.41 | 20.74 (4.14 to 47.61) | -0.92 (-1.73 to -0.06) |
| tAD 3 |  |  |  | 1 | 0.58 | 28.78 (8.59 to 56.30) | -0.62 (-1.37 to 0.16) | 0 | 0.41 | 20.74 (4.14 to 47.61) | -0.92 (-1.73 to -0.06) |
| lvPPA 1 |  | 5.43 (4.29) | 9 | 4 | 0.76 | 38.01 (15.97 to 63.66) | -0.33 (-1.00 to 0.35) | 2 | 0.47 | 23.51 (6.20 to 49.07) | -0.80 (-1.54 to -0.02) |
| tAD 1 | RAVLT Recognition (30) | 18.60 (2.45) | 8 | 20 | 0.61 | 69.67 (42.12 to 90.43) | 0.57 (-0.20 to 1.31) | 26 | **0.02** | 98.76 (90.46 to 100.00) | 3.02 (1.31 to 4.70) |
| tAD 2 |  |  |  | 17 | 0.56 | 27.89 (8.03 to 55.39) | -0.65 (-1.40 to 0.14) | 17 | 0.56 | 27.89 (8.03 to 55.39) | -0.65 (-1.40 to 0.14) |
| tAD 3 |  |  |  | 16 | 0.35 | 17.53 (2.75 to 43.73) | -1.06 (-1.92 to -0.16) | 18 | 0.82 | 41.24 (17.35 to 68.03) | -0.25 (-0.94 to 0.47) |
| lvPPA 1 |  | 24.10 (2.54) | 9 | 27 | 0.31 | 84.48 (60.48 to 97.58) | 1.14 (0.27 to 1.97) | 18 | **0.05** | 2.61 (0.01 to 14.44) | -2.40 (-3.71 to -1.06) |
| tAD 1 | ROCF Copy (36) | 29.40 (8.49) | 8 | 32 | 0.78 | 60.95 (33.94 to 84.31) | 0.31 (-0.41 to 1.01) | 35 | 0.55 | 72.32 (44.79 to 92.10) | 0.66 (-0.13 to 1.41) |
| tAD 3 |  |  |  | 36 | 0.49 | 75.64 (48.27 to 94.01) | 0.78 (-0.04 to 1.56) | 20.5 | 0.36 | 17.81 (2.86 to 44.08) | -1.05 (-1.09 to -0.15) |
| lvPPA 1 |  | 30.30 (7.05) | 9 | 33 | 0.73 | 63.71 (37.94 to 85.34) | 0.38 (-0.31 to 1.05) | 34 | 0.63 | 68.40 (42.44 to 88.70) | 0.53 (-0.19 to 1.21) |
| lvPPA 2 |  |  |  | 31 | 0.93 | 53.64 (28.83 to 77.51) | 0.10 (-0.56 to 0.75) | 14 | 0.06 | 2.98 (0.02 to 15.68) | -2.31 (-3.58 to -1.01) |
| lvPPA+ 2 |  | 20.40 (15.50) | 8 | 36 | 0.37 | 81.29 (54.72 to 96.76) | 1.01 (0.12 to 1.85) | 35 | 0.40 | 79.80 (52.96 to 96.11) | 0.94 (0.07 to 1.76) |
| tAD 1 | ROCF Immediate Recall (36) | 6.28 (6.05) | 8 | 15.5 | 0.19 | 90.30 (67.58 to 99.45) | 1.52 (0.46 to 2.55) | 20 | 0.07 | 96.50 (81.43 to 99.98) | 2.27 (0.89 to 3.60) |
| tAD 3 |  |  |  | 4 | 0.73 | 36.66 (13.93 to 63.88) | -0.38 (-1.08 to 0.36) | 3.5 | 0.68 | 33.91 (11.99 to 61.33) | -0.46 (-1.18 to 0.29) |
| lvPPA 1 |  | 13.28 (7.13) | 9 | 20 | 0.40 | 80.13 (55.03 to 95.71) | 0.94 (0.13 to 1.72) | 14 | 0.93 | 53.70 (28.88 to 77.46) | 0.10 (-0.56 to 0.75) |
| lvPPA 2 |  |  |  | 18.5 | 0.51 | 74.65 (48.86 to 92.73) | 0.73 (-0.03 to 1.46) | 5.5 | 0.33 | 16.55 (2.82 to 40.79) | -1.09 (-1.91 to -0.23) |
| lvPPA+ 2 |  | 3.12 (2.89) | 8 | 6.5 | 0.31 | 84.67 (59,06 to 98.04) | 1.17 (0.23 to 2.06) | 0.5 | 0.42 | 21.06 (4.29 to 47.98) | -0.91 (-1.72 to -0.05) |
| tAD 1 | ROCF Delayed Recall (36) | 5.19 (6.36) | 8 | 11.5 | 0.38 | 80.97 (54.33 to 96.63) | 0.99 (0.11 to 1.83) | 18.5 | 0.09 | 95.54 (78.63 to 99.96) | 2.09 (0.79 to 3.35) |
| tAD 3 |  |  |  | 2 | 0.65 | 32.55 (11.05 to 60.03) | -0.50 (-1.22 to 0.25) | 2 | 0.65 | 32.55 (11.05 to 60.03) | -0.50 (-1.22 to 0.25) |
| lvPPA 1 |  | 12.00 (7.48) | 9 | 18 | 0.47 | 76.57 (50.97 to 93.85) | 0.80 (0.02 to 1.54) | 14 | 0.81 | 59.70 (34.24 to 82.27) | 0.27 (-0.41 to 0.93) |
| lvPPA 2 |  |  |  | 15 | 0.71 | 64.33 (38.52 to 85.79) | 0.40 (-0.29 to 1.07) | 4 | 0.34 | 17.01 (3.01 to 41.37) | -1.07 (-1.88 to -0.22) |
| lvPPA+ 2 |  | 1.44 (2.27) | 8 | 4.5 | 0.24 | 87.78 (63.50 to 98.93) | 1.35 (0.35 to 2.30) | 0 | 0.57 | 28.44 (8.37 to 55.96) | -0.63 (-1.38 to 0.15) |
| tAD 1 | ROCF Recognition (24) | 17.90 (3.14) | 8 | 21 | 0.38 | 80.78 (54.12 to 94.55) | 0.98 (0.10 to 1.82) | 22 | 0.26 | 87.03 (62.38 to 98.74) | 1.30 (0.32 to 2.24) |
| tAD 3 |  |  |  | 12 | 0.12 | 6.04 (0.12 to 25.16) | -1.87 (-3.04 to -0.67) | 14 | 0.28 | 14.07 (1.56 to 39.14) | -1.24 (-2.15 to -0.28) |
| lvPPA 1 |  | 18.90 (1.63) | 9 | 20 | 0.54 | 73.00 (47.11 to 91.73) | 0.68 (-0.07 to 1.39) | 19 | 0.95 | 52.26 (27.62 to 76.25) | 0.06 (-0.59 to 0.71) |
| lvPPA 2 |  |  |  | 18 | 0.61 | 30.73 (10.72 to 56.70) | -0.55 (-1.24 to 0.17) | 15 | **0.05** | 2.65 (0.01 to 14.57) | -2.39 (-3.70 to -1.06) |
| lvPPA+ 2 |  | 17.40 (2.50) | 8 | 21 | 0.22 | 89.16 (65.66 to 99.24) | 1.44 (0.40 to 2.43) | 11 | **0.05** | 2.33 (0.003 to 14.43) | -2.56 (-4.03 to -1.06) |

Significant two-tailed probabilities are indicated in bold red font. A composite recognition score was calculated for RAVLT and ROCF in the same way: the sum of true positives and true negatives (i.e., maximum of 30 for RAVLT and 24 for ROCF). The score for Trail Making Test was calculated as the ratio of correct lines to total time in seconds to complete the test. While the overall group sample sizes were nine, ten, and nine for tAD, lvPPA, and lvPPA+, respectively, we included a column for “Group N” as a few patients did not complete some tests, affecting the sample size for those tests. ACE-R, Addenbrooke’s Cognitive Examination – Revised; CAT, Comprehensive Aphasia Test; CSB, Cambridge Semantic Battery; lvPPA, logopenic variant primary progressive aphasia; RAVLT, Rey Auditory Verbal Learning Test; ROCF, Rey Osterrieth complex figure; SD, standard deviation; tAD, typical Alzheimer’s disease.

**
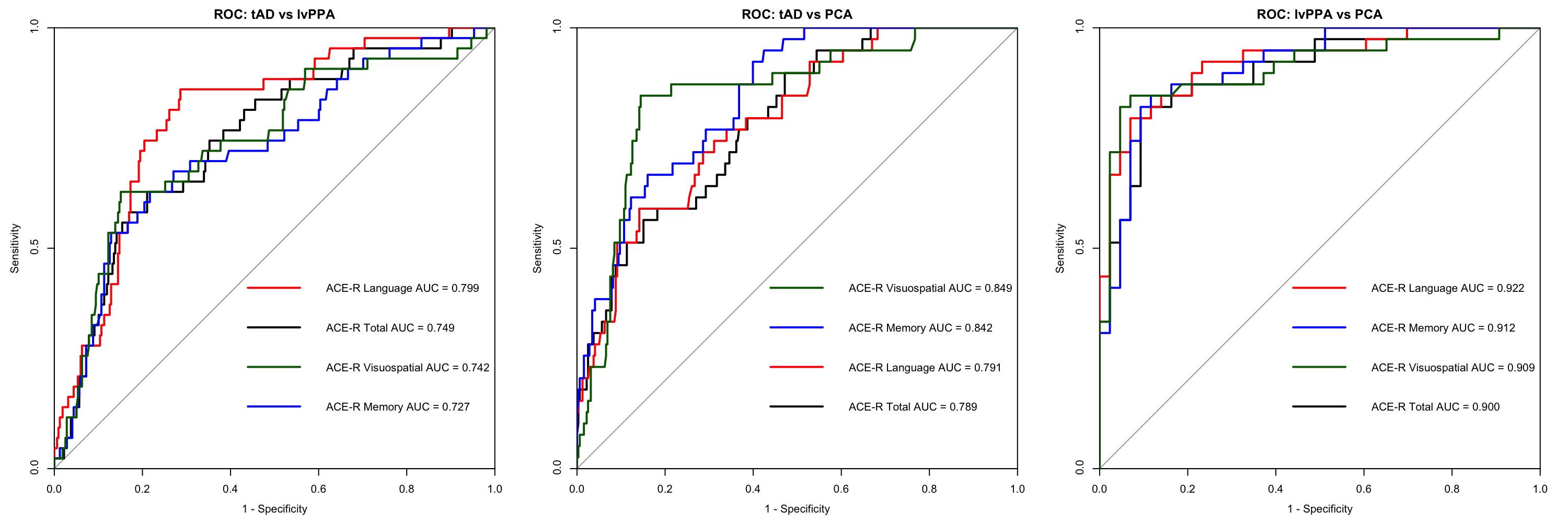
Supplementary Figure 1. Receiver operating characteristic curves distinguishing between retrospective patient groups (left: tAD versus lvPPA; middle: tAD versus PCA; right: lvPPA versus PCA).** Binary logistic regressions were conducted for each pairwise group comparison (i.e., tAD vs. lvPPA; tAD vs. PCA; lvPPA vs. PCA). In each model, the primary predictor was ACE-R subdomain score of interest (i.e., language, memory, visuospatial) or the total ACE-R score, with age and sex included as covariates.


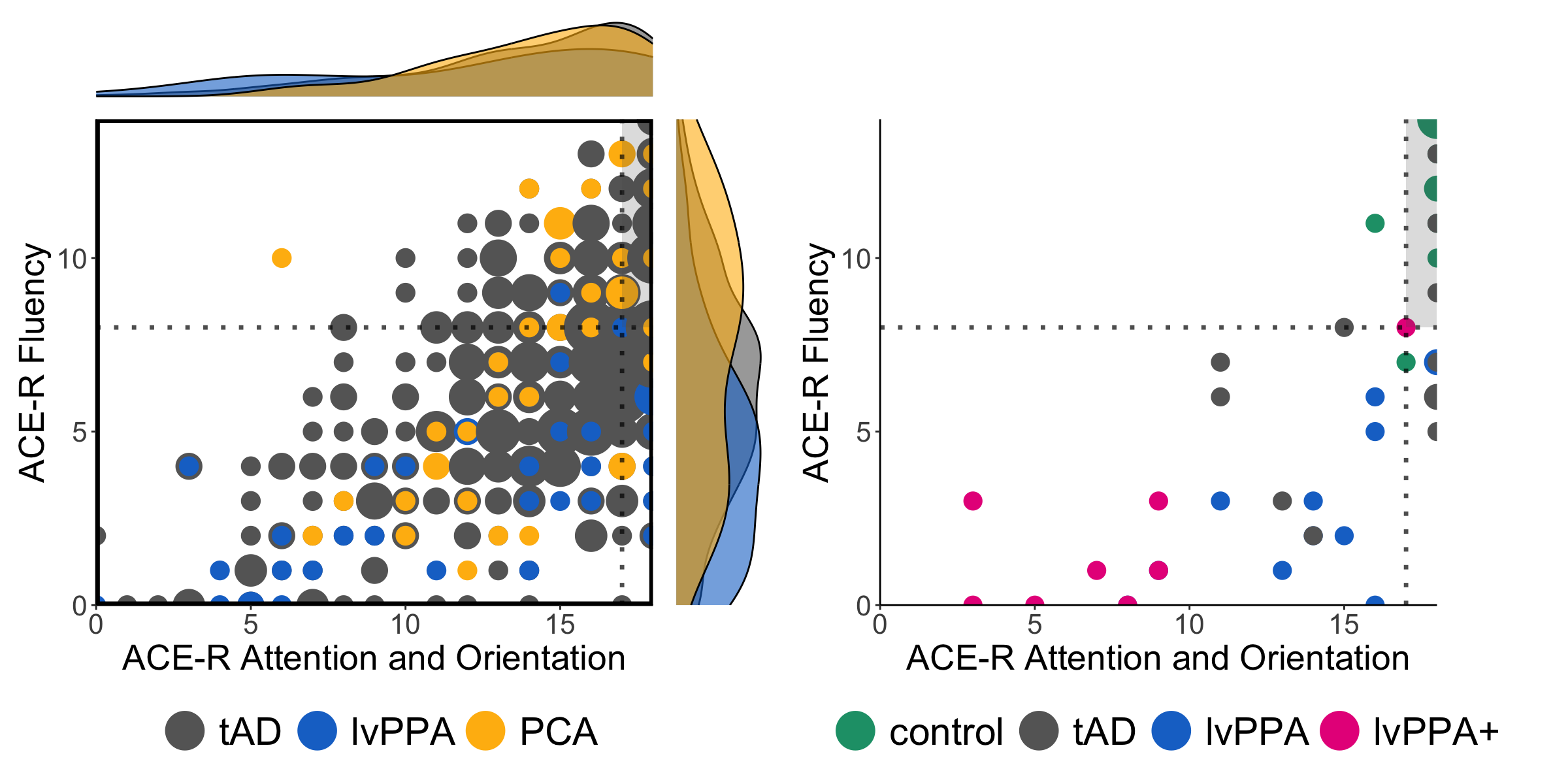


**Supplementary Figure 2. Scatterplots of ACE-R fluency and attention/orientation domains over the study population (left: control *n* = 12, tAD *n* = 9, lvPPA *n* = 10, lvPPA+ *n* = 8) and research participants who were classified as having an AD clinical phenotype from the memory clinics of the Cambridge University Hospital (right: AD *n* = 329, lvPPA *n* = 44, PCA *n* = 40).** The dotted lines represent the ACE-R cutoff scores from published healthy control normative data ^28^ and the shaded grey sections portray the quadrant of normality. In the scatterplot on the right, the larger dots portray more participants who had the same scores and the accompanying raincloud plots on the sides show the density curves of the data distribution per group.

**
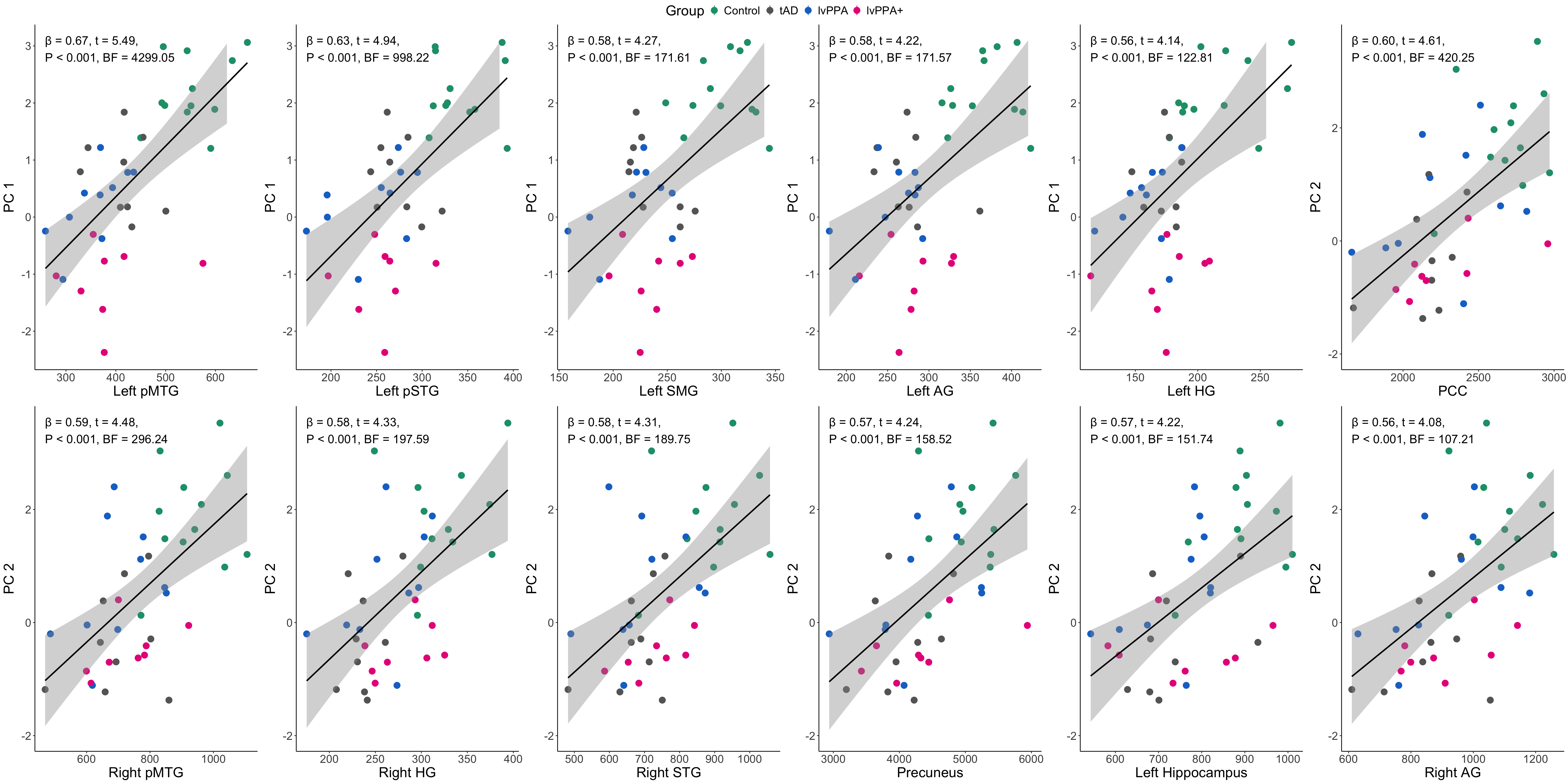
 Supplementary Figure 3. Associations between principal component (PC) scores and region of interest (ROI) grey matter volumes across the whole group that showed extreme Bayesian evidence.** Only results showing extreme Bayesian evidence (BF > 100) are shown. AG, angular gyrus; BF, Bayes factor; HG, Heschl’s gyrus; PCC, posterior cingulate cortex; pMTG, posterior middle temporal gyrus; pSTG, posterior superior temporal gyrus; SMG, supramarginal gyrus.


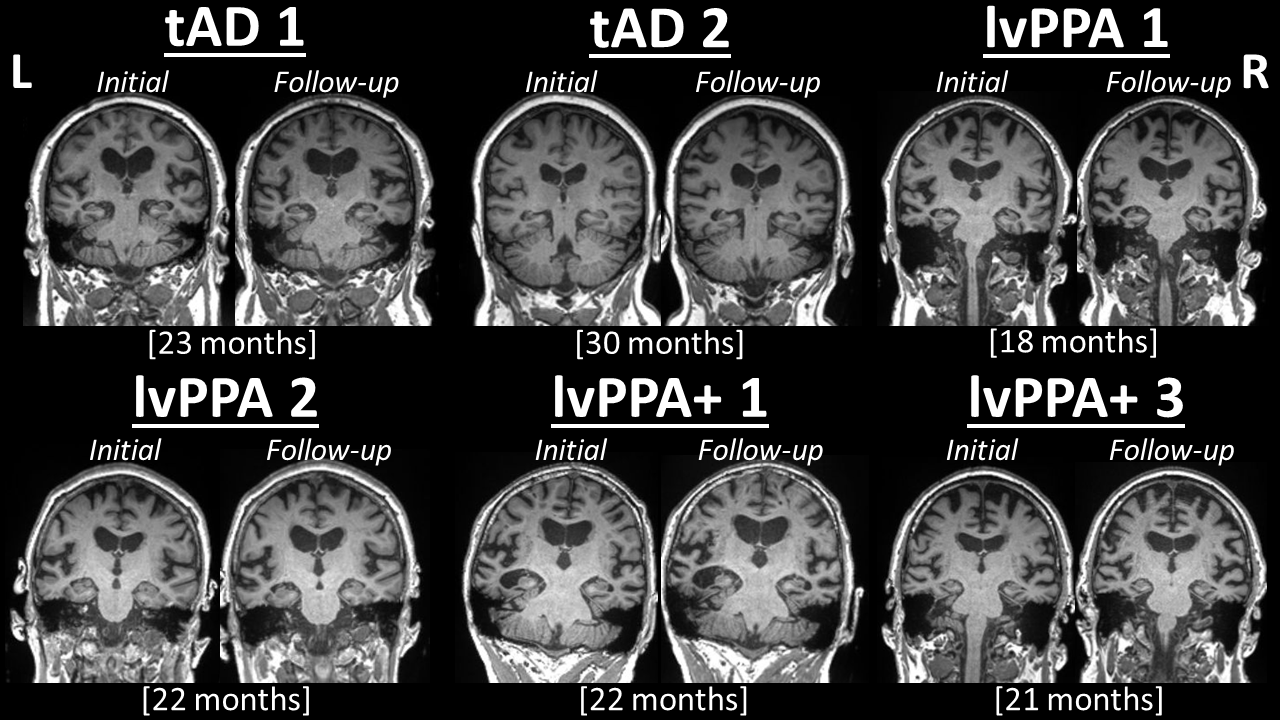


**Supplementary Figure 4. Neuroimaging for the longitudinal patients.** Coronal MRI slices at initial and follow-up MRI scans are shown. Time since initial scan is indicated as months in square brackets.
